# Supplementary material for: MetaRibo-Seq measures translation in microbiomes
Source: Nat Commun. 2020 Jun 29;11:3268. doi: 10.1038/s41467-020-17081-z (PMC7324362; doi:10.1038/s41467-020-17081-z)
Supplement: Supplementary file 10 — Supplementary Data 7 [file 41467_2020_17081_MOESM10_ESM.zip › File2/Confidence_High_Taxonomy/132878_out.krona.html]

Javascript must be enabled to view this page.

members
magnitude
magnitudeUnassigned
count
unassigned
taxon
rank

132878\_out

6

6
superkingdom
2

6
phylum
1224

class
6
1236

order
6
91347

family
6
543

6
genus
561

562
species

SRS012273\_contig\_number\_contig-100\_147.277227SRS076876\_contig\_number\_274SRS077646\_contig\_number\_contig-100\_566.567SRS144362\_contig\_number\_36509SRS146888\_contig\_number\_contig-100\_762.93504SRS147022\_contig\_number\_7780
6
